# Modeling and Performance of the IEEE 802.11p Broadcasting for Intra-Platoon Communication

**DOI:** 10.3390/s18092971

**Published:** 2018-09-06

**Authors:** Chong Yu, Shuaizong Si, Hongye Guo, Hai Zhao

**Affiliations:** School of Computer Science and Engineering, Northeastern University, Shenyang 110819, China; guohongye_neu@163.com (H.G.); zhaoh@mail.neu.edu.cn (H.Z.)

**Keywords:** platoon, intra-vehicle communication, M/G/1/K queuing theory, Markov process, performance analysis

## Abstract

Road capacity, traffic safety, and energy efficiency can be extremely improved by forming platoons with a small intra-vehicle spacing. Automated controllers obtain vehicle speed, acceleration, and position through vehicular ad hoc networks (VANETs), which allows the performance of platoon communication to make a significant impact on the stability of the platoon. To the best of our knowledge, there is not much research relating to packet delay and packet dropping rate of platoon communication based on the IEEE 802.11p broadcasting. In this paper, we introduce platoon structure model, vehicle control model, and communication model for a single platoon scenario. By utilizing Markov process and M/G/1/K queuing theory, we put forward an analytical model to assess the property of intra-vehicle communication. The analytical model is validated by simulations and the influence of communication parameters on intra-vehicle communication performance are discussed. In addition, the experimental results demonstrate that the IEEE 802.11p-based intra-vehicle communication guarantee the stability of platoon.

## 1. Introduction

With the support of cooperative adaptive cruise control (CACC) for automated driving and the assist of vehicle ad-hoc network (VANETs) for intra-vehicle communication, a series of succeeding vehicles form a platoon [1,2]. Driving vehicles in platoon patterns brings many benefits [3,4,5]. Road capacity can be enhanced while traffic jams can be lessened considerably because each vehicle moves at a constant speed and maintains a constant small intra-vehicle distance ahead. Besides, since the streamlining of a platoon can diminish air drag, the platoon pattern can decrease both energy consumption and exhaust emissions. Furthermore, with the assistance of advanced technologies, it is considered that driving in a platoon pattern is more secure and more comfortable.

The Federal Communications Commission allocated 75 MHz of bandwidth in the 5.9-GHz band for Dedicated Short Range Communications (DSRC) to assist intelligent transportation system (ITS), such as platoon. The overall bandwidth is divided into seven 10-MHz channels including one control channel (CCH) and six service channels (SCHs). Safety-related messages (e.g., road warning, vehicle speed, and vehicle acceleration) only are transmitted on CCH, while non-safety information (e.g., weather condition, media access, and mobile Office) are conveyed on SCHs. In present IEEE standards, the only standard medium access protocol for VANETs is IEEE 802.11p. This protocol was presented based on the existing 802.11 standard to meet requirements of Vehicle To Vehicle (V2V) and Vehicle To Infrastructure (V2I) communications in a high mobility VANETs environment. The broadcast communication model is widely applied in V2V communications to notify the neighbor vehicle of safety-related messages because of highly dynamic topology and low delay constraints in VANETs. There are many studies focusing on the communication scheme [6,7,8,9]. Those are presented based on IEEE 802.11p and aiming to achieve low dissemination delay and improve the VANETs performance.

The core of platoon control is to ensure the stability of platoon, i.e., spacing errors do not amplify as they propagate upstream from one vehicle to another [10]. The preceding vehicle’s speed, position, and acceleration sent through intra-platoon communication are adopted as inputs in vehicle controllers to achieve simultaneous stabilization of vehicles of platoon. The primary assumption holding in the previous studies focuses on platoon control is that the vehicle controllers acquire timely and accurate information [11,12,13]. However, many published studies have indicated that the communication performance has a significant effect on vehicle control [14,15,16,17]. The increases of communication delay and packet dropping rate lead to a decrease of the safety and comfort of the platoon. Inspired by this, many studies have been conducted on platoon control considering the effect of communication. Ploeg et al. proposed a novel control and scheduling algorithm considering fixed communication delay [18]. Guo et al. investigated issues of communication and control of a platoon limited by capacity constraint and packet dropping in VANETs environment [19]. Segata et al. introduced independent Bernoullian random losses and analyzed the impact of the packet error rate on the failure rate of the maneuver, proved that packet loss mainly affect the maneuver from a coordinating point of view [20]. However, these approaches are not perfect, because the time-varying delay and packet loss rate are caused by rapid changes in network topology, the condition of communication channels, and the mobility of vehicles in platoon. Therefore, it is necessary to investigate the communication process of platoon and analyze its performance.

Our work concentrates on the performance analysis of the IEEE 802.11p broadcast which is used to convey control messages on the CCH for intra-vehicle communication under a VANETs environment. The contributions of our work can be described as follows:

1. Firstly, we establish a communication model of intra-platoon communication in a single platoon scenario. The intra-vehicle communication process is divided into two part: back-off process of channel competition for transmitting control messages and queuing process of packets in buffers. The back-off process of broadcasting is modeled using a 1-D Markov chain and the queuing process is established exploiting M/G/1/K queuing theory.
2. Secondly, we consider an unsaturated and imperfect condition in our analytical model. The theoretical solution of packet delay and packet dropping rate are derived considering different factors together, including the imperfect wireless channels, various platoon size, distribution of packet generation rate.
3. Thirdly, results have been provided in a single platoon scenario under a wide range of settings. The accuracy of the analytical model is evaluated. The impacts of bit error rate, packet generation rate, and platoon size on the intra-vehicle communication performance are investigated. The effect of intra-platoon communication performance on the stability of platoon is discussed.

The manuscript is organized as follows: Section 2 overviews the related research on performance analysis of vehicle communication in VANETs. In Section 3, the concept of platoon including platoon structure model, vehicle control model and communication model are introduced. In Section 4, we present our performance analysis approach in detail. The experiments, relevant results, and analysis are shown and discussed in the following Section 5. Section 6 summarizes the conclusions and future work.

## 2. Related Works

The main technique for the 802.11 MAC is the distributed coordination function (DCF). Many studies analyze the performance of VANET based on 802.11 DCF. Peng et al. [21] presented a probabilistic analytical model of IEEE 802.11p DCF under a saturated condition. In [22], they derived performance metrics employing a performance analysis of IEEE 802.11p DCF under an unsaturated condition. However, these two studies focus on inter-platoon communications rather than intra-platoon communication. A multi-priority Markov model is derived for investigating the change of the platoon throughput with the connectivity probability [23], but it does not analyze the delay and packet dropping rate. The analytical formulations developing by Du [24] has estimated messages propagation delay via VANETs, but vehicles on roads do not form a platoon. Jia et al. [25] developed a 4-D Markov chain to model the cooperative retransmission process to discuss delay and uplink throughput of drive-thru Internet. The deficiency of this paper is that the assumed communication network is saturated and the packet loss of platoon communication is not taken into account.

Some analytical studies investigate the communication performance of V2V communication based on the IEEE 802.11 broadcast. Hafeez [26] analyzed the successful reception ratio, conflict probability, and throughput of IEEE 802.11p broadcast without queuing process. An improved analytical model supporting two access modes was established by Han et al. [27] to assess the throughput of IEEE 802.11p EDCA. Song et al. [28] extended the existing model by taking the character of multichannel switching, virtual collision, and AIFS differentiation into account. Han’s model only considered a saturated condition, while Song’s method supported both saturated and unsaturated conditions. Although Fernandes [29] obtained delay distribution of typical scenes by implementing an experiment platform for platoon of inter-vehicle communication enabled autonomous vehicles in Matlab, the corresponding theoretical research is absent. Yang et al. [30] proposed an analytical model and established a periodic broadcast scheme for VANETs. Yang’s work makes an improvement by applying freezing back-off time counter and a D/M/1 queue. Considering the MAC queue with a finite capacity, two Markov chain models for ACs with different priorities were proposed by Yao et al. [31] to analyze the reliability of the safety-related information propagated on the CCH. However, no channel errors were considered by Yang et al. and Yao et al.

These existing studies have significantly promoted the development of platoon; nevertheless, the value of these studies in practical application is degraded because the treatment methods of platoon communication model and performance are too idealistic or not comprehensive. Furthermore, there is not sufficient study focusing on the quantitative analysis for inter-vehicle communication process and performance of platoon. Motivated by the aforementioned points, our work concentrates on communication process modeling and communication performance metrics analysis of IEEE 802.11p in a single platoon scene. Taking back-off process of channel competition and queuing process in buffers into account. Moreover, we take an unsaturated and imperfect condition into design consideration, including the imperfect wireless channels, various platoon size, and distribution of packet generation rate.

## 3. Platoon System

### 3.1. Platoon Structure Model

**Figure 1:**
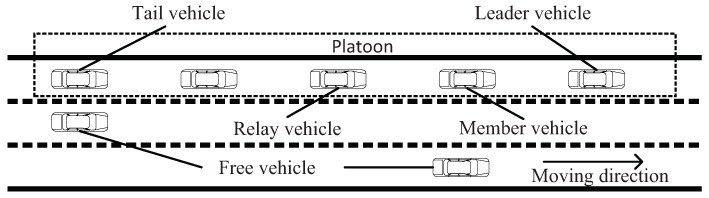
Platoon structure model.

According to the spatial position and functionalities, vehicles in a platoon can be classified into five roles: leader vehicle, tail vehicle, member vehicle, relay vehicle, and free vehicle. The structure of platoon is shown in Figure 1.

(1) Leader vehicle: It refers to the first vehicle in a platoon. A leader vehicle is responsible for establishing and administering the platoon with the coordinate of Advanced Traffic Management System, e.g., controlling driving behavior of the vehicle in a platoon, collecting data from other vehicles on the road, and identifying and broadcasting the information of platoon. The movement of the leader vehicle is the reference movement for all remaining vehicles in the platoon to follow.
(2) Tail vehicle: It locates at the end of a platoon. The tail vehicle is an essential hub for inter-platoon communication and is responsible for establishing a connection with the next platoon.
(3) Member vehicle: It is a vehicle within the platoon that locates neither at the begin nor the end of a platoon. A member vehicle following a specified control algorithm receives messages from the leader vehicle and its preceding vehicle.
(4) Relay vehicle: Any member vehicles in a platoon can act as a relay vehicle, which is in charge of assisting leader vehicle in disseminating messages to all vehicles in a platoon.
(5) Free vehicle: Vehicles that do not belong to any platoon are called free vehicles. A free vehicle sends a request to the leader vehicle of a platoon when it wants to join a platoon. After obtaining the permission of the leader vehicle, the free vehicle can perform join operation.

In a platoon, the behavior of a vehicle (such as acceleration/deceleration and joining or leaving a platoon) not only depends on the driver’s wishes but also obey management and constraint from platoon control center. The leader vehicle is generally regarded as the control center. A member vehicle transfers request messages to the leader vehicle within the same platoon when the driver wants to change driving behavior according to its own needs, e.g., destination and rest. Then, the leader vehicle makes a judgment based on the current traffic situation. If the leader vehicle responds to the member vehicle’s request, all vehicles in the platoon have to adjust to maintain the stability of the platoon.

### 3.2. Vehicle Control Model

The vehicle dynamics are highly nonlinear. However, under certain assumptions [32] and with appropriate feedback, vehicle dynamics can be linearized. We apply a simple vehicle dynamics model for a vehicle’s longitudinal motion as in [33], which can be described as a function. The spacing error is defined as

(1) $$\epsilon_{i}=p_{i-1}-p_{i}-l_{i-1}-g_{i-des}$$

where $p_{i}$ represents the position of the $i^{\mathrm{th}}$ vehicle; $p_{i-1}$ and $l_{i-1}$ denote position and length of its preceding vehicle, respectively; and $g_{i-des}$ is the desired gap between $i^{\mathrm{th}}$ and ${\left(i-1\right)}^{\mathrm{th}}$ vehicle. Starting at t=0 with initial condition $\epsilon_{i}\left(0\right)$, the goal of consensus control is to achieve convergence

(2) $$\epsilon_{i}\left(t\right)\to 0,\;as\;t\to \infty$$

assuming that $\epsilon_{i}=0$, we can obtain the desired position of the $i^{\mathrm{th}}$ vehicle

(3) $$p_{i-des}=p_{i-1}-l_{i-1}-g_{i-des}$$

Considering feedback messages includes relative acceleration, speed, and position of both preceding vehicles and the leader vehicle, vehicles’ desired acceleration is expressed as

(4) $$u_{i-des}=\left(1-q_{1}\right)a_{i-1}+q_{1}a_{l}-q_{2}\left(v_{i}-v_{i-1}\right)-q_{3}\left(v_{i}-v_{l}\right)-q_{4}\epsilon_{i}$$

where $q_{1}$, $q_{2}$, $q_{3}$ and $q_{4}$ are design parameters. Here, variables with symbol “*l*” refer to leader vehicles. A first-order filter is used to model the actuator lag and signal processing delay.

(5) $$u_{i-des}=\left(1+\mu s\right)u_{i}$$

where μ is the total delay including control delay, sensor detection and processing delays, and actuator delays, which is treated as a constant.

### 3.3. Communication Model

Intra-vehicle communication is important for a platoon to maintain stability, which is due to the need for frequent and reliable exchange of information between vehicles in a platoon. The core of platoon communication is V2V communication. In this work, we adopt DSRC to support the V2V communication. The 75 MHz band allocated for DSRC is divided into one CCH and six SCHs. The control channel is responsible for transmitting safety information, while service channels are used for non-safety applications. Broadcast mode based on IEEE 802.11p is applied in platoon communication. IEEE 802.11p is the only standard medium access control protocol for VANETs [34]. There are several differences between broadcast mode and traditional unicast mode. Firstly, to avoid an acknowledgment (ACK) storm, each vehicle in a platoon does not need to transmit an ACK to the origin vehicle when it receives information. Secondly, although there is a failed transmission in broadcast mode, the source vehicle would never retransmit the lost packets. Compared with unicast communications, broadcast communication has a higher transmission failure probability. Lastly, the overhead of RTS/CTS access mode weakens the performance of systems with high mobility, such as platoon. Thus, the RTS/CTS handshake is not applied in broadcast model.

**Figure 2:**
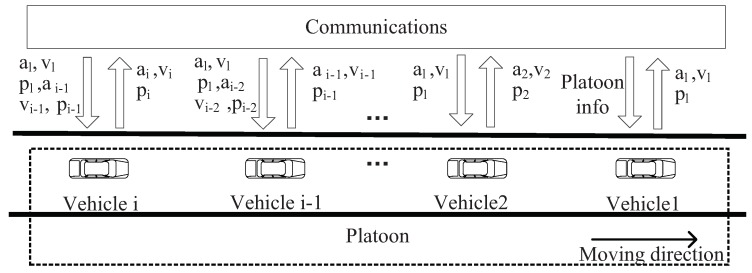
Platoon communication framework.

The platoon communication framework is shown as Figure 2. Since a vehicle’s longitudinal motion is noticeably affected by the mobility of the leader vehicle, we must ensure that the information of the leader vehicle can be received by each vehicle in a platoon. To simplify the communication model, we assume the length of platoon does not exceed the communication range *R* of a leader vehicle, which indicates the size of platoon is restricted. Vehicles in a platoon are equipped with a transceiver used for sand and receive messages. A leader vehicle can send information to any vehicles within a platoon, while member vehicles only send information to its following vehicle. In terms of contents and functionalities, messages transmitted in platoons are classified into two types: control information and non-control information. The control information includes vehicle driving behavior, traffic conditions, and accident warning; the non-control information includes application information shared among vehicles, such as media, entertainment, and office services. The control information flow among vehicles can significantly affect the stability and security of a platoon. Therefore, we focus on the transmission process of the control information. Note that we establish our analytical model without considering priority since all safety messages have the same priority.

## 4. Analytical Model

### 4.1. Intra-Platoon Back-Off Process

In this section, we analyze the performance of IEEE 802.11p broadcast for intra-platoon communication in a single platoon scenario. In IEEE 802.11p broadcast mode, vehicles with fresh information to be transmitted monitor the channel states and use back-off mechanism to compete for channels. Before the back-off process, one vehicle needs to choose a random number within [0,CW] as the back-off time. Once the initial contention window CW is defined, it will keep constant for all back-off processed because there is no retransmission in the broadcast mode. The state of back-off time counter can be classified into three categories: (1) The back-off time counter is reduced by one as long as the channel is detected idle; (2) The back-off counter is frozen when a packet is transmitted on the channel; (3) If the channel is detected idle again over a DIFS, the back-off counter is reactivated. When a vehicle’s backoff-time counter reaches zero, the vehicle instantly broadcasts the information.

In the analysis, we make the following hypotheses: ideal channel conditions (i.e., no hidden terminals and capture); vehicles do not have packets available for transmission at all time, which indicates the data traffic condition is unsaturated; and there are channel impairments under a practical channel condition, so a packet can encounter transmission errors.

We assume that the bit error rate is $p_{b}$. Then, $p_{e}$ the transmission error probability of packers caused by channel error can be expressed as

(6) $$p_{e}=1-{\left(1-p_{b}\right)}^{L}$$

and $L=H_{phy}+H_{mac}+D_{l}$ is the valid data length. $D_{l}$ denotes data size, $H_{phy}$ represents the size of data in PHY and $H_{mac}$ represents the size of data in MAC. Packets sent by a vehicle can be received by all vehicles in the same platoon due to vehicles in a platoon are within the communication range of each other. Thus, the probability of channel congestion can be computed as

(7) $$p_{p}=1-{\left[1-\left(1-q_{em}\right)p\right]}^{N-1}$$

where *N* represents the platoon size (vehicles per platoon), and $q_{em}$ denotes new packets arrival probability. According to $p_{p}$ and $p_{e}$, the probability of unsuccessful transmission can be expressed as

(8) $$\begin{array}{c} p_{f}=1-\left(1-p_{p}\right)\left(1-p_{e}\right)=p_{p}+p_{e}-p_{p}p_{e} \end{array}$$

We define $p_{na}$ as the arrival rate of a new packet, and $q_{em}$ as the probability that a vehicle does not send data packets in a slot time. Then, the probability that a channel is detected as idle in the back-off process can be represented as $q_{em}^{N}$. When a vehicle’s sending queue is not empty, no matter how many packets there are in the sending buffer, the vehicle will contend for the channel in the same way according to the IEEE 802.11p broadcast. Stochastic process b(t) and s(t) are defined as the back-off time counter and the back-off order for a certain vehicle, respectively. Then, {s(t),b(t)} is modeled with a 1-D Markov-chain. Let $b_{r}=\lim_{t\to \infty }P\left\{b\left(t\right)=r\right\}$, 0≤r≤CW represent the stationary distribution, and $b_{N}$ be the empty state of buffer. According to the Markov-chain model, we have

(9) $$\left\{\begin{array}{c} b_{r}=\frac{\left(b_{0}+b_{N}\right)p_{na}}{\left(CW+1\right)q_{em}^{N}}+b_{r+1},\;r=0,1,\cdots ,CW-1\\ b_{r}=\frac{\left(b_{0}+b_{N}\right)p_{na}}{\left(CW+1\right)q_{em}^{N}},\;r=CW\\ \sum_{k=0}^{CW}b_{r}+b_{N}=1 \end{array}\right.$$

Solving Equation (9), the transmission attempt probability of packets in unsaturated flow condition is given as follow

(10) $$p=b_{0}=\frac{2q_{em}^{N}p_{na}}{CWp_{na}+2q_{em}^{N}}$$

### 4.2. MAC Layer Queuing Process Model

According to [34], the control message generation rate is dynamically adjusted. It is related to the road condition, vehicle behavior, and traffic condition. If there is an increase in the vehicle movement dynamics, the packets arrival rate increase to ensure the movement dynamic is correctly reflected in the message. Thus, the transmission of messages generated by a vehicle in a platoon can be regarded as a random event in practice. We assume the probability of packets arrival conforms to the Poisson distribution with a parameter of λ, where λ represents the number of packets arriving in a unit time. When a vehicle has packets to transmit, these packets will be sent to the queue buffer. Packets wait in the buffer according to the principle of first in first out. Certain packet access the communication resources when it reaches to the head of the queue. M/G/1/K queuing theory is applied to simulate the queuing process, and the maximum length of a queue is *K*.

The time from channel competition to successful transmission or unsuccessful transmission can be defined as the packet service time [35], which generally set as integer times of the idle slot. We define the service time distribution as $p_{st}^{T}$, $T\in [0,T_{m}]$. The maximum service time is $T_{m}\tau$, where τ is the free slot. Following [36], we define X(t),(t≥0) as the state of the queuing system at time *t*. Let $\delta_{j}$ be the time instance of the $j^{th}$ packet departure. We consider the embedded Markov process $\left\{X_{j}\right\}$, where $X_{j}$ is the state of the queuing system just before $\delta_{j}$. The state space of the embedded Markov process can be represented as

(11) $$S^{\prime }=\left\{A_{0},A_{1},A_{2},\cdots ,A_{K}\right\}$$

It should be noted that $A_{K}$ indicates K−1 packets wait in the queue and one is serviced. When the size of buffer *K* is set to be one, the state space of the embedded Markov process only contains two states: $S^{\prime }=\left\{A_{0},A_{1}\right\}$. $A_{0}$ is the state that the queue is emptied and no packet is serviced and $A_{1}$ represents the state that no packets wait in the queue and one packet is serviced [31]. In this condition, a new incoming packet is immediately served without waiting.

Define $a_{k}$ as the probability that *k* packets arrive during one packet service time. As the packet arrival is a Poisson process with rate λ, we have

(12) $$a_{k}=\sum_{T=0}^{T_{m}}p_{st}^{T}\frac{{\left(\lambda T\tau \right)}^{k}}{k!}e^{-\lambda T\tau }$$

We suppose $p_{st}^{T}=0$ represents no packets arrival. Then, the probability that the sending buffer of a communication vehicle has new packets arrival in a slot time equals

(13) $$p_{na}=1-e^{-\lambda T_{idle}}$$

where $T_{idle}$ is the average idle time of the vehicle buffer when the channel is busy.

(14) $$\begin{array}{cc} T_{idle} & =\;\tau {\left[1-\left(1-q_{em}\right)p\right]}^{N-1}+T_{c}\{{\left[\left(1-q_{em}\right)p\right]}^{N-1}\\ & -\;\left(N-1\right)\left(1-q_{em}\right)p{\left[1-\left(1-q_{em}\right)p\right]}^{N-2}\}\\ & +\;T_{e}p_{e}\left(N-1\right)\left(1-q_{em}\right)p{\left[1-\left(1-q_{em}\right)p\right]}^{N-2}\\ & +\;T_{s}\left(1-p_{e}\right)\left(N-1\right)\left(1-q_{em}\right)p{\left[1-\left(1-q_{em}\right)p\right]}^{N-2} \end{array}$$

In Equation (14), $T_{s}$ and $T_{e}$ are the length of time of successful and collided packet transmission, respectively. $T_{s}$ and $T_{e}$ can be expressed as follows

(15) $$T_{e}=T_{s}=DIFS+H_{phy}+H_{mac}+D_{l}$$

In addition, $T_{c}$ is the time consumed by the channel conflict in the process of data transmission and can be calculated as

(16) $$T_{c}=DIFS$$

Let $p_{k}^{que}\left(0\leq k\leq K\right)$ be the steady-state probability that $X_{j}=A_{k}$, which is

(17) $$p_{k}^{que}=\left\{\begin{array}{c} p_{0}^{que}a_{k}+\sum_{i=1}^{k+1}p_{i}^{que}a_{k-i+1},\;k=0,1,\cdots ,K-1\\ p_{0}^{que}\left(1-\sum_{l=0}^{k-1}a_{l}\right)+\sum_{i=1}^{k}p_{i}^{que}\left(1-\sum_{l=i-1}^{k-1}a_{l-i+1}\right),\;k=K \end{array}\right.$$

According to M/G/1/K model, the probability that a vehicle has not transmitted data packets in a slot time can be derived is

(18) $$q_{em}=\frac{p_{0}^{que}}{p_{0}^{que}+\lambda {\bar{T}}_{sv}}$$

where $\lambda {\bar{T}}_{sv}$ denotes the probability of the channel being occupied, and ${\bar{T}}_{sv}$ is the average packet service time.

**Figure 3:**
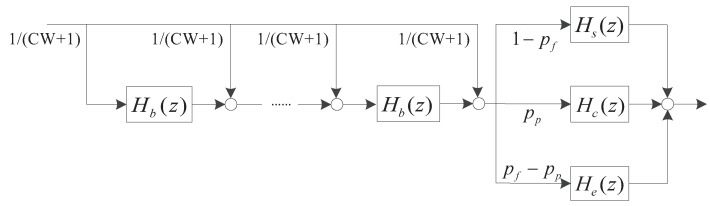
MAC service time linear system diagram.

We can then apply the transfer-function approach, in which the packet transmission process is characterized by a linear system [37], as shown in Figure 3. Binary exponential back-off is performed when the channel is detected idle. A packet is sent after a back-off procedure, the probability that packets collision is $p_{p}$, and the probability of frame error is $p_{f}-p_{p}$, and the probability that packets successful transmission is $1-p_{f}$. Each stage is considered as a subsystem of the service system, and the delay *t* generated in the time domain is represented by transfer function $Z^{t}$ in the *Z*-domain. The service system is the cascade of multi-subsystems which means that system output is the superposition of multiple delay factors $Z^{t}$. The transfer function of the packet transmission process is derived as

(19) $$\begin{array}{cc} H(z) & =\left[p_{p}H_{c}\left(z\right)+\left(p_{f}-p_{p}\right)H_{e}\left(z\right)+\left(1-p_{f}\right)H_{s}\left(z\right)\right]\frac{1}{CW+1}\sum_{n=0}^{CW}H_{b}^{n}\left(z\right) \end{array}$$

where $H_{c}\left(z\right)=z^{T_{c}}$ represents the time distribution transfer function of packets transmission failure caused by transmission conflict. $H_{s}\left(z\right)=z^{T_{s}}$ and $H_{e}\left(z\right)=z^{T_{s}}$ represent the time distribution transfer function of successful packets delivery and the time distribution transfer function of packets transmission failure due to channel errors, respectively. The time distribution transfer function of the back-off procedure $H_{b}\left(z\right)$ is given by

(20) $$\begin{array}{cc} H_{b}\left(z\right) & ={\left[1-\left(1-q_{em}\right)p\right]}^{N-1}z^{\tau }+{\left[1-\left(1-q_{em}\right)p\right]}^{N-2}\left(N-1\right)\left(1-q_{em}\right)pz^{T_{s}}\\ & +\left\{1-{\left[1-\left(1-q_{em}\right)p\right]}^{N-1}-{\left[1-\left(1-q_{em}\right)p\right]}^{N-2}\left(N-1\right)\left(1-q_{em}\right)p\right\}z^{T_{c}} \end{array}$$

In Equation (20), the coefficient of $z^{\tau }$ and $z^{T_{c}}$ are the probability of channel idle and channel conflict. While the coefficient of $z^{T_{s}}$ represents the probability that one of the N−1 vehicles transfer packets successfully. The back-off time is uniformly chosen in the range [0,CW] where CW is the size of the contention window. When the channel is idle in a slot time, we can postulate that the back-off counters of all nodes decrease by one. The state of channels is divided into three: (1) idle; (2) busy; and (3) transmission successful. Thus, the back-off time is the mean value of the unit slot, conflict consumption time and successful transmission time. According to packets transmission process, the probability generating function of service time distribution can be expressed as $H\left(z\right)=\sum_{T=0}^{T_{m}}p_{st}^{T}z^{T\tau }$. Combined with Equations (18) and (19), discrete probability $p_{st}^{T}$ can be obtained.

### 4.3. Performance Measures

In this section, packet dropping rate and delay are calculated based on our analytical model. The delay in the whole process includes queuing delay and back-off delay. In this paper, the time of transmitting packets to the recipient after successful access channel is ignored. Based on the average queue size and packet arrival rate, we can derive the queuing delay

(21) $$\begin{array}{cc} T_{que} & =\frac{\sum_{i=0}^{K-1}\frac{ip_{i}^{que}}{\lambda {\bar{T}}_{sv}+p_{0}^{que}}+K\left(1-\frac{1}{\lambda {\bar{T}}_{sv}+p_{0}^{que}}\right)}{\lambda /(\lambda {\bar{T}}_{sv}+p_{0}^{que})}\\ & =K\frac{\lambda {\bar{T}}_{sv}+p_{0}^{que}-1}{\lambda }+\sum_{i=0}^{K-1}\frac{ip_{i}^{que}}{\lambda } \end{array}$$

where ${\bar{T}}_{sv}=\frac{dH(z)}{dz}{|}_{z=1}=\sum_{T=0}^{T_{m}}p_{st}^{T}\cdot T\tau$.

In the case of successful transmission, the delay in the back-off process of the packet competitive channel is

(22) $$T=\frac{CW}{2}T_{slot}$$

(23) $$\begin{array}{cc} T_{slot} & =\tau {\left[1-\left(1-q_{em}\right)p\right]}^{N}+T_{c}\left\{1-{\left[1-1\left(1-q_{em}\right)p\right]}^{N}-N\left(1-q_{em}\right)p{\left[1-\left(1-q_{em}\right)p\right]}^{N-1}\right\}\\ & +\;T_{e}p_{e}N\left(1-q_{em}\right)p{\left[1-\left(1-q_{em}\right)p\right]}^{N-1}+T_{s}\left(1-p_{e}\right)N\left(1-q_{em}\right)p{\left[1-\left(1-q_{em}\right)p\right]}^{N-1} \end{array}$$

Thus, the total delay of information transmission in a platoon is

(24) $$T_{delay}=T+T_{que}$$

Meanwhile, packet dropping rate equals with the probability of packets transmission failure, and it can be expressed as

(25) $$P=p_{p}+p_{e}-p_{p}p_{e}$$

To obtain delay and packet dropping rate of platoon communication, we assume that vehicles always have packets to be sent, i.e., $q_{em}=0$. By solving Equations (6)–(8) and (10), we can get *p* and $p_{f}$. Then, $p_{st}^{T}$, $p_{k}^{que}$ and $q_{em}$ can be obtained by solving Equations (16), (17) and (19). If $q_{em}$ is greater than 1 × 10${}^{-6}$ (the set threshold), we update *p* and $p_{f}$ to the probability in the condition of buffer may be empty. Otherwise, we do not need to update *p* and $p_{f}$ because the possibility of buffer vacant is very low. Then, we can obtain delay and packet dropping rate by substituting Equations (22) and (23) into Equation (24).

## 5. Simulations Verification

We verified our analytical model using computer simulation and investigated the communication performance under various configurations of $p_{b}$, λ, and *N*. We used the open source vehicular network simulation framework Veins which is frequently used in academic research. It consists of two parts: the mobility characteristic of vehicles was modeled using SUMO and the communication networking characteristic was modeled using OMNeT++.

**Figure 4:**
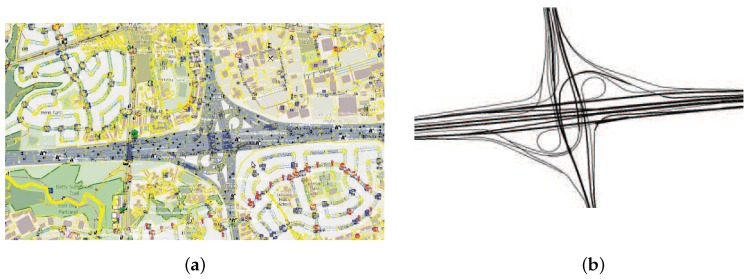
Simulation scenario: (**a**) real map; and (**b**) road topology.

The simulation scenario is based on a real map of the city of Shenyang in China, which is shown in Figure 4. Figure 4a shows a real map extracted from OpenStreetMap and Figure 4b illustrates the road topology without buildings. The size of the scenario is 1 km × 1.6 km. The number of vehicles in a platoon varied within the interval [2, 10]. The speed of vehicles was set to be 60 km/h based on the typical motorways speed limitation. Many DSRC studies have employed 6 Mb/s as the data rate since it seems to be a good choice for safe message transmission in most cases. As a result, 6 Mb/s was selected in our simulations. As for wireless communication simulation, propagation signal modeling is one of the most fundamental issues. The Nakagami model has proven to be the best fading radio propagation model for simulation of a VANET environment. Consequently, we adopted this propagation model in our experiments. Transmission power was 20 dBm and noise-floor was −89 dBm. The values of other parameters are listed in Table 1. The parameters were chosen from the latest draft IEEE 802.11p standard. To guarantee the accuracy of the experimental results, we repeated each independent experiment thirty times.

### 5.1. Model Verification

**Figure 5:**
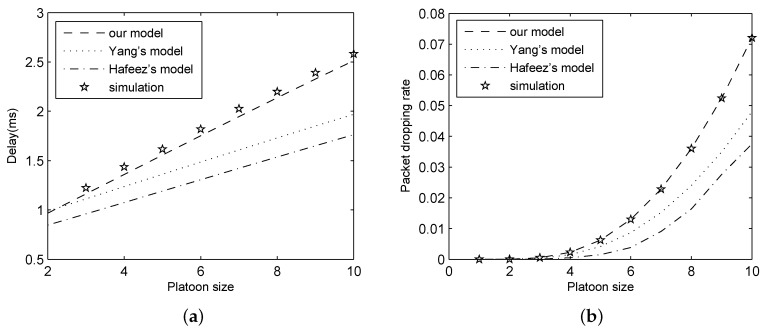
Results for different analytical models: (**a**) delay; and (**b**) packet dropping rate.

The proposed analytical model was validated in the above mentioned environment and compared with Yang’s model and Hafeez’s model. In these experiments, we set λ=100, $p_{b}=$ 1 × 10${}^{-5}$ and K=20. The simulation and analytical results for delay and packet dropping rate are shown in Figure 5.

In Figure 5, we can observe that the results of our analytical model perfectly match that of experiment, while those of Yang’s model and Hafeez’s model have a great gap with the simulation results. This suggests that our analytical model is more exact compared with two existing models. This is because we not only consider the queuing process, but also take channel errors into consideration in the modeling process.

### 5.2. The Effect of Bit Error Rate

**Figure 6:**
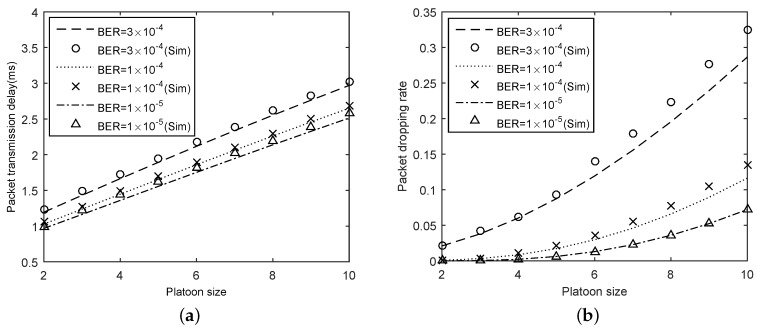
Results for varying $p_{b}$: (**a**) delay; and (**b**) packet dropping rate.

To evaluate the effect of the channel errors, we considered a case that λ=100 and K=20. In the comparison, $p_{b}=$ 1 × 10${}^{-5}$, 1 × 10${}^{-4}$, and 3 × 10${}^{-4}$, respectively. The results are shown in Figure 6. In general, our analytical model is quite accurate since the results of analytical results and that of experiments are similar for different bit error rates.

Figure 6a depicts the impact of bit error rates on delay. We can observe that the delay increased with the rise of $p_{b}$ for a given *N*. Moreover, the delay increased with the growth of the platoon size. This is because in larger platoons, more packets need to be sent in a buffer. However, these packets must wait longer in the buffer before being transmitted. In addition, more packets competing for channels leads to higher conflicts. Figure 6b shows that the packet dropping rate against the platoon size under different bit error rates. Rising bit error rate affects packet dropping rate in the negative direction. For example, the packet dropping rate is 0.05 when $p_{b}$ = 1 × 10${}^{-5}$ and N=10, and the packet dropping rate is 0.33 under the condition of $p_{b}=$ 3 × 10${}^{-4}$ and N=10. Besides, by increasing the platoon size, the probability of collision grows during the packet transmission, which causes a higher packet loss rate for delivered packets.

### 5.3. The Effect of Packet Arrival Rate

**Figure 7:**
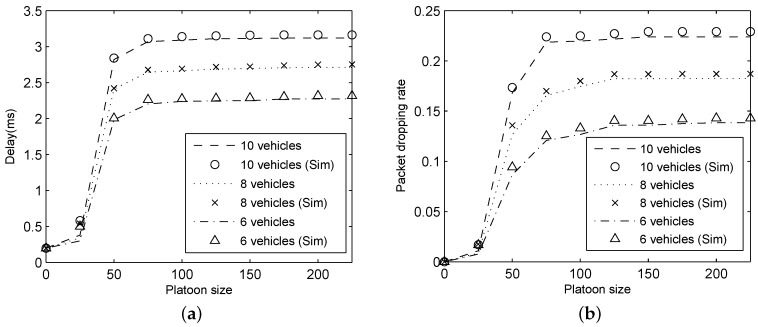
Results for varying λ: (**a**) delay; and (**b**) packet dropping rate.

To study the effect of packet arrival rate on platoon communication performance, we change the platoon size and packet arrival rate λ. In these experiments, we set $p_{b}=$ 1 × 10${}^{-5}$ and K=20. The simulation and analytical results for delay and packet dropping rate are depicted in Figure 7.

As shown in Figure 7, delay and packet dropping rate calculated based on our analytical model perfectly match simulation results for different packet arrival rate. An observation from Figure 7a is that the increase of packet arrival rate causes the delay to rise. Regarding packet dropping rate, as plotted in Figure 7b, the packet dropping rate is increasing with the growth of packet arrival rate for a fixed platoon size because the probability of unsuccessful transmission increases as the traffic load rises. Meanwhile, in Figure 7, the performance metrics rapidly increase under the non-saturated condition (λ≤100). With the growth of λ, the network will reach saturation condition finally (λ>100) and then the performance metrics is constant.

### 5.4. The Stability of Platoon

Finally, the effect of performance metrics on platoon stability is evaluated. The term “string stability” is usually utilized interchangeably with “platoon stability” in the study of platoon, which suggests any non-zero acceleration, velocity, and position changes of vehicles in a platoon do not amplify when they propagate upstream, i.e., $∥\epsilon_{2}{∥}_{\infty }\;>\;∥\epsilon_{3}{∥}_{\infty }\;>\;\cdots \;>\;∥\epsilon_{n}{∥}_{\infty }$.

We set up the following simulation scenario: A platoon with eight vehicles is placed on a straight one-way road, and the length of the road is 5000 m. According to [38], $q_{1}=0.34$, $q_{2}=1.2$, $q_{3}=0.53$, and $q_{4}=0.6$. The standstill spacing is 10 m and the vehicle length is 5 m. Moreover, λ=150, $p_{b}=$ 3 × 10${}^{-4}$ and K=20. The leader vehicle starts with an initial velocity of 30 m/s and maintains this velocity until t=5 s. Meanwhile, member vehicles and the tail vehicle have a fixed velocity of 30 m/s, and the spacing between any neighborhood is also fixed. At time t=5 s, the leader vehicle accelerate with an acceleration of 2.5 m/s${}^{2}$ until the velocity of leader vehicle reaches to 55 m/s. At time t=25 s, we let the leader vehicle decelerate with acceleration 5 m/s${}^{2}$ until the velocity of the leader vehicle reaches to 5 m/s.

**Figure 8:**
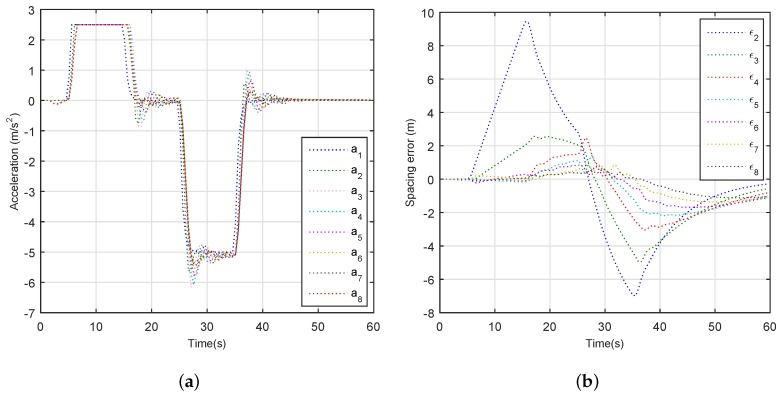
Vehicle dynamic characteristics: (**a**) acceleration; and (**b**) spacing error.

Figure 8 depicts the vehicle’s behavior when the leader vehicle accelerates or decelerates. The results demonstrate that accelerations and spacing errors of the seven following automated vehicles in the platoon gradually decline upstream, and the string stability is guaranteed perfectly, which indicates excellent tracking performance of the following automated vehicles with the transmission delay and packet dropout. Thus, it suggests that the IEEE 802.11p-based communication can support the timely delivery of vehicle information among platoons for diverse on-road applications.

### 5.5. Simulations Summary

Form the above-mentioned experimental results, five conclusions can be drawn. (a) Due to the consideration of the queuing process and channel errors, our analytical model accurately reflects the performance of platoon communication; (b) Reducing bit error rate is an effective method to improve the performance of intra-vehicle communication; (c) Under the premise of the safety of platoon, system performance can be improved by decreasing packet arrival rate; (d) The rise of platoon size has a negative effect on the performance of platoon communication, so a large platoon size is inappropriate; (e) The IEEE 802.11p broadcast communication satisfies the requirements of timely and reliable transmissions of platoon in practice. It is undeniable that there are some minute differences between the results of simulation experiments and that of real scenarios. This phenomenon is attributed to the factors in two aspects. Firstly, the interference of free vehicles in platoon communication is ignored in our simulation experiments, while the interference is unavoidable in real scenarios. Secondly, platoon communication includes two parts: inter-platoon communication and intra-platoon communication. However, the impact of inter-platoon communication is not taken into account in simulation scenarios. Therefore, simulation results are slightly lower than real results.

## 6. Conclusions and Future Work

In this work, an analytical model is proposed to estimate the performance of the unsaturated IEEE 802.11p broadcast networks over an imperfect channel for intra-vehicle communication. Markov chain model is adopted to describe back-off mechanism for broadcast, and M/G/1/K theory is adopted to describe queuing process in MAC layer. Specifically, analytical expressions for delay and packet dropping rate are derived accordingly. Experiment results are shown to prove the perfection of the analytical model and to investigate the effect of bit error rate, packet arrival rate, and platoon size on the performance of intra-vehicle communication. The following conclusions can be drawn from the results. There is no benefit to provide a large bit error rate and packet arrival rate. Communication performance is gradually weakening with the increase of platoon size. We can limit the platoon size, the bit error rate, and the packet arrival rate to satisfy the reliability and timeliness requirements of information transmission for platoon.

In future work, our team will concentrate on relay-based multi-hop communication model for multi-platoon. In addition, how the inter-vehicle dynamic communication model influences the control algorithm of platoon should be discussed.

## Figures and Tables

**Table 1:** Simulation parameters.

Parameter	Value	Parameter	Value
*R*	300 m	$T_{m}$	5000
$D_{l}$	4096 bits	τ	20 μs
$H_{mac}$	224 bits	CW	15
$H_{phy}$	192 bits	DIFS	64 μs
$H_{ack}$	112 + 192 bits	Data rate	6 Mbps
